# MEMS Biomimetic Acoustic Pressure Gradient Sensitive Structure for Sound Source Localization

**DOI:** 10.3390/s90705637

**Published:** 2009-07-15

**Authors:** Peng An, Weizheng Yuan, Sen Ren

**Affiliations:** Micro and Nano Electro Mechanical System Laboratory, Northwestern Polytechnical University, Xi’an City, Shaanxi Province, 710072, China; E-Mails: anpeng@mail.nwpu.edu.cn (P.A.); rensen@mail.nwpu.edu.cn (S.R.)

**Keywords:** MEMS, biomimetic, parasitoid fly, pressure gradient, sound source localization

## Abstract

The parasitoid fly *Ormia ochracea* shows an astonishing localization ability with its tiny hearing organ. A novel MEMS biomimetic acoustic pressure gradient sensitive structure was designed and fabricated by mimicking the mechanically coupled tympana of the fly. Firstly, the analytic representation formulas of the resultant force and resultant moment of the incoming plane wave acting on the structure were derived. After that, structure modal analysis was performed and the results show that the structure has out-of-phase and in-phase vibration modes, and the corresponding eigenfrequency is decided by the stiffness of vertical torsional beam and horizontal beam respectively. Acoustic-structural coupled analysis was performed and the results show that phase difference and amplitude difference between the responses of the two square diaphragms of the sensitive structure are effectively enlarged through mechanical coupling beam. The phase difference and amplitude difference increase with increasing incident angle and can be used to distinguish the direction of sound arrival. At last, the fabrication process and results of the device is also presented.

## 1. Introduction

**Figure 1.**
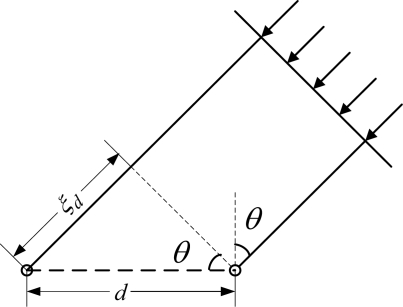
Schematic diagram of acoustic array with two elements.

For most of animals, the only cues available for localizing auditory stimulus are minute interaural intensity difference (IID) and interaural time difference (ITD), in other words, the intensity difference and TOA (Time Of Arrival) difference between the acoustic signals received by the ear near and the ear far from the sound source [1]. The physics of sound propagation imposes fundamental constraints on sound localization: for a given frequency, the smaller the receiver, the smaller the available cues [2]. Since sound wave intensity attenuates with increasing distance, the smaller the distance between two ears, the smaller intensity difference. For TOA difference, this can be explained through the simplest array with only two elements as shown in Figure 1. The array element is ordinary acoustic pressure sensor. If the distance between the two elements is *d*, the incident angle of incoming plane wave (the angle between the direction of sound propagation and the normal direction of the array) is *θ*, and the array is in the far-field of sound source, the TOA difference *τ* between the acoustic signals received by the two elements can be represented as:

(1) $$\tau =\frac{\xi_{d}}{c_{0}}=\frac{d\;\text{sin}\;\theta }{\lambda f}$$

and the phase difference *ϕ* between the acoustic signals received by the two elements can be represented as:

(2) $$\phi =2\pi \;f\;\tau =2\pi \;f\frac{d\;\text{sin}\;\theta }{c_{0}}=2\pi \frac{d}{\lambda }\;\text{sin}\;\theta$$

Here *ξ_d_* is the acoustic path difference, *f* is the frequency of the sound, *c*_0_ is the sound speed, and *λ* is the wavelength of acoustic wave. As we can see from Formula (1), the direction of sound arrival *θ* can be derived from the TOA or phase difference, and each difference directly depends on the ratio of array elements distance to sound wavelength. The smaller the distance between two array elements (ears), the smaller TOA or phase difference. This is also the reason why it is difficult to miniaturize acoustic sensor array for sound source localization.

Owing to the aforementioned reasons, for small insects with the distance between its two ears being very small relative to the acoustic wavelength, the interaural intensity and TOA differences can be extremely small for sound source localization. But the parasitoid fly *Ormia ochracea*, with its left and right tympana only 450 to 520 μm apart from each other shows a remarkable ability to localize sound sources [3]. Supposing the two tympana are independent of each other, the maximal time difference of the acoustic signal reaching the two tympana can be calculated at around 1–2 μs using Formula (1). For such minute time differences, research has shown it is insufficient for reliable neural encoding of directional information [4]. But in fact, the gravid female fly must find and deposit her parasitic larvae on a singing field cricket. Its surprising localization ability lies in its special hearing organ that utilizes an intertympanal bridge between its two tympana. Although the amplitude difference or phase difference between the acoustic signals received by the right and the left tympana is very tiny, the amplitude difference or phase difference between the mechanical responses of the left and the right tympana is efficiently enhanced owing to mechanical coupling between the two tympana, so that these enlarged cues can be used for sound source localization [3,5,6]. Actually, the fly has a localization ability that approximates that of humans [7,8].

The special minute hearing organ and the astonishing localization ability of this parasitoid fly give us an inspiration to design a miniature acoustic sensor for sound source localization using MEMS technology. In order to enlarge the cues for sound source localization, the hearing organ makes a response to acoustic pressure gradient as well as mean acoustic pressure. When it comes to engineering design, acoustic sensor with its output response depending on the direction of acoustic wave propagation also requires the sensing of acoustic pressure gradient. The hearing organ of the parasitoid fly gives us a biological prototype to mimic. A MEMS biomimetic acoustic pressure gradient sensitive structure for sound source localization is designed and characterized in the following sections of the article.

## 2. Structure Design and Analytical Analysis

### 2.1. Biomimetic Principle

**Figure 2.**
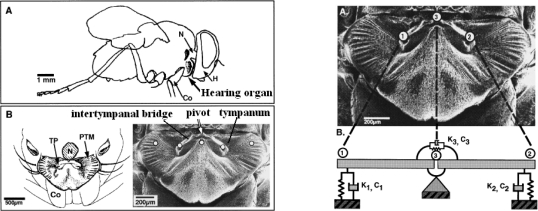
Hearing organ of parasitoid fly *ormia ochracea* and its mechanical model.

The fly’s left and right tympana are not physically separated, but are contained within a common air-filled chamber, a mere 450 to 520 μm apart from each other. The fly’s hearing organ as well as its mechanical model is shown in Figure 2 [3]. This anatomy structure results in minuscule interaural time difference and no measurable interaural intensity difference.

In fact, the two tympana are connected to each other and to the pivot point through a cuticular structure, the intertympanal bridge. All of these form a structure that has two special vibration modes: in-phase mode with both tympana moving in the same direction with equal amplitude. This mode is also called as bending mode named from bending vibration of the intertympanal bridge; out-of-phase mode with both tympana moving in opposite direction with equal amplitude. This mode is also called as rocking mode named from structure’s rotating vibration around the pivot. The response of the structure in the acoustic field will be the combination of these mode responses. With these two special vibration modes and appropriated mechanical properties of the hearing organ, the combination results in constructive superposition of the mode responses in one tympanum and destructive superposition of the mode responses in another tympanum. In other words, mechanical response of the tympanum near the sound source is enhanced, meanwhile, that of the tympanum farther from the sound source is weakened. As a result, both interaural amplitude difference and interaural phase difference are effectively enlarged. According to the fly’s position relative to the sound source, the interaural amplitude difference and interaural phase difference will be different, so that the direction of incident wave can be determined.

### 2.2. Structure Design

**Figure 3.**
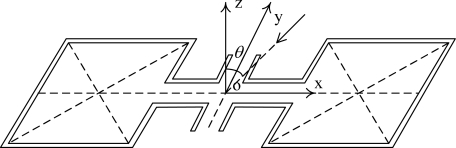
Schematic diagram of the sensitive structure.

As we can see from aforementioned biomimetic principle: the mechanical coupling of the two tympana through the intertympanal bridge is the fundamental of the fly’s ability to localization; the out-of-phase mode response is crucial to enlarge the interaural difference. The out-of-phase mode response, that is, structure’s rotating vibration around the pivot, can be regarded as structure’s response to the moment originating from acoustic pressure gradient. For such reason, it also can be said that the localization ability of the parasitoid fly lies in its hearing organ’s high sensitivity to the pressure gradient. A biomimetic acoustic pressure gradient sensitive structure is design based on the foregoing biomimetic principle and the feasibility of micromachining process by mimicking the mechanically coupled tympanums of the fly, as shown in Figure 3 schematically:

The structure is separated from the surroundings with narrow slits. Free diaphragm edges can increase the structural response to pressure gradients [9]. The width of the slits is small enough so that the incident acoustic wave almost cannot go through the slits and acts on the backside of the diaphragm within the frequency range of interest [10,11]. The surface layer of the structure is connected square diaphragms that mimic the mechanically coupled tympana of the parasitoid fly’s hearing organ. The horizontal (the direction of *x* axis) beam under the diaphragm, which mimics intertympanal bridge, realizes the mechanically coupling of the two square diaphragms. The whole structure is suspended by the vertical (the direction of *y* axis) beam under the diaphragm. The beam also provides restoring torsional moment during the structure vibration. Stiffeners are designed under the diaphragm to enhance the stiffness of the diaphragm to avoid unnecessary diaphragm deformation without heavily increasing the mass of it. In order to increase the moment acting on the structure resulting from incident acoustic wave, the distance between the two square diaphragms should be increased. By detecting the displacement of each diaphragm through capacitive or optical methods, the incident acoustic wave can be measured.

### 2.3. Resultant Normal Force and Resultant Moment

A cartesian coordinate system was created as shown in Figure 3, with the origin labeled *o* locates at the geometry center of the surface layer of the structure, *x* axis along the direction of horizontal beam, *y* axis along the direction of vertical beam, *z* axis perpendicular to the surface layer of the structure.

The diaphragm is in the *xoy* plane and the direction of wave propagation is parallel to *xoz* plane. The angle between the direction of wave propagation and the normal direction of the diaphragm is *θ*. The incidence harmonic wave can be expressed in the following form:

(3) $$p=p_{a}e^{j(\omega t+\mathrm{kx}\;\text{sin}\;\theta +\mathrm{kz}\;\text{cos}\;\theta )}$$

where *P_a_* is the amplitude of sound pressure, *i* is the imaginary unit, *ω* is the angular frequency of sound, *t* is the time, *k* is the wave number, *k* = *ω/c*_0_ *=* 2π*/λ*, *x*, *z* is the x and z coordinates of a point in the sound field.

As we can see from Formula (3), the amplitude of sound pressure acting on each point of the diaphragm is the same, but the phase of sound pressure is different according to position. In another words, owing to the existing of x, z component of sound pressure gradient, the transient sound pressure is different at the same moment according to its x and z coordinates. The effects of incidence wave can be equivalent to resultant normal force that bends the structure and resultant moment that forces the structure rotating around vertical beam. To simplify the derivation process, some reasonable approximation is made. As known from acoustic principle, the sound pressure upon the diaphragm should be the superposition of incidence wave and scattering wave, but when the length of structure is much smaller than the wavelength, the effects of scattering can be ignored, and then the sound pressure upon diaphragm is approximately the same as the pressure of incident wave [12]. Besides that, because of the width of horizontal beam and vertical beam is much smaller than the diaphragm, the force acting on them is also ignored.

If the width of each square diaphragm is *a*, the distance between two square diaphragms is 2*L*, the resultant force *F* acting on the structure can be expressed as:

(4) $$F=\int_{s}\mathrm{pdS}=\frac{{2\mathrm{ap}}_{a}e^{j\omega t}}{k\;\text{sin}\;\theta }\left\{\text{sin}[k(L+a)\text{sin}\;\theta ]-\text{sin}(\mathrm{kL}\;\text{sin}\;\theta )\right\}$$

and the resultant moment *M* can be expressed as:

(5) $$\begin{array}{l} M=\int_{s}\mathrm{xpdS}=\frac{{2\mathrm{ap}}_{a}e^{j(\omega t-\pi /2)}}{k\;\text{sin}\;\theta }•\{\text{cos}[k(L+a)\;\text{sin}\;\theta ](L+a)-\text{cos}(\mathrm{kL}\;\text{sin}\;\theta )L-\\ \text{sin}[k(L+a)\;\text{sin}\;\theta ]/\left(k\;\text{sin}\;\theta \right)-\text{sin}(\mathrm{kL}\;\text{sin}\;\theta )/\left(k\;\text{sin}\;\theta \right)\} \end{array}$$

**Figure 4.**
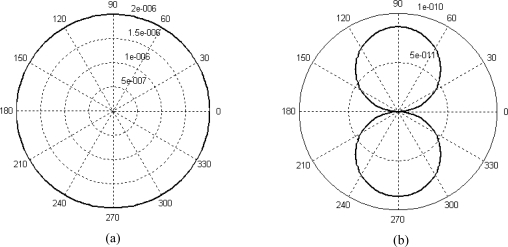
Resultant force (a) and resultant moment (b) versus incident angle.

It is clear that the resultant force and resultant moment also vary harmonically with time. If *L* is equal to 0.4 mm, *a* is equal to 1 mm, sound frequency *f* is equal to 1 kHz and the sound pressure amplitude *p_a_* is equal to 1 Pa, the relation curves between the amplitude of resultant force or resultant moment and the incident angle can be plot in polar coordinate system, as shown in Figure 4.

As we can see from the figure, when the characteristic length of the structure is much smaller than the wavelength, the resultant force keep constant as the incident angle varies. There is approximately no relationship between the resultant force and incident angle. But the resultant moment expresses a directivity pattern of “∞” as the incident angle varies. In order to enhance the directivity of the structure, the sensitivity to pressure gradient should be increased.

## 3. FEA Simulation

It is difficult to characterize the dynamic behavior of the sensitive structure in the acoustic field through analytic method because of complicated structure vibration equation and distributed acoustic loading. For such reason, finite element analysis was performed using FEA software ANSYS.

### 3.1. Mode Analysis

Firstly, structure modal analysis was performed to determine the vibration characteristics (natural frequencies and mode shapes) of the sensitive structure. During the simulation, besides in-phase and out-of-phase vibration modes, several other vibration modes were also found within the frequency range of analysis; for example, the vibration mode in which the structure rotates around *z* axis and bending vibration mode of each diaphragm itself. Displacement measurement of the diaphragm will become difficult and less accurate if these additional vibration modes coexist in the working frequency range. Since only out-of-phase mode or both in-phase and out-of-phase modes can be utilized to achieve different directivity pattern, other additional vibration mode responses should be suppressed by choosing appropriate structural parameters. Stiffeners under the diaphragm are introduced to increase the stiffness of each diaphragm itself. The bending stiffness along *x* direction of the vertical torsional beam and the bending stiffness along *y* direction of the horizontal beam should also be sufficiently high. The purpose of all these design consideration is to increase the eigenfrequency of additional vibration modes so as to they are much higher than the upper limit of the working frequency range.

**Figure 5.**
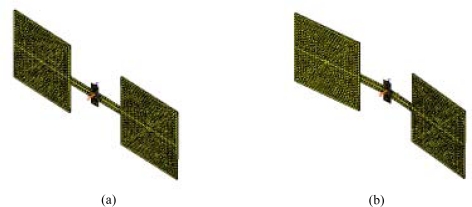
First (a) and second (b) mode shapes of the sensitive structure.

Following structural parameters are used for FEA analysis: Young’s modulus of the silicon material is 169 Gpa, Poisson’s ratio is 0.23, the width of each square diaphragm is 800 μm, the thickness of the diaphragm is 2μm, the distance between the two square diaphragms is 800 μm, the width of horizontal beam is 80 μm and the width of the vertical beam is 15 μm. Acquired first and second mode shapes are shown in Figure 5.

In the first vibration mode, the structure rocks around the vertical torsional beam. Excepting the vertical beam, the other parts of the structure behave just like a rigid body. The vibration phase difference of the left and right diaphragm equals *π*, in another words, they are in antiphase. The mode is just the aforementioned out-of-phase mode.

In the second vibration mode, both the left and right diaphragms move towards the same direction at the same moment and the horizontal beam is bended. The vibration phase of the left and right diaphragm is always equal. The mode is just the aforementioned in-phase mode.

**Figure 6.**
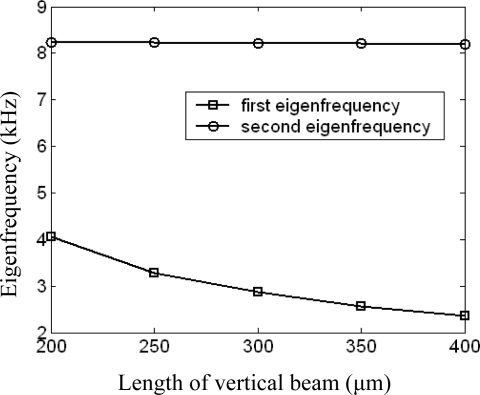
Length of vertical beam versus first and second eigenfrequency.

For a given horizontal beam length, the relationship curve between the length of vertical beam and the first and second eigenfrequency is shown in Figure 6. As we can see from the figure, the first eigenfrequency decreases with the length of vertical beam increasing and the second eigenfrequency keeps almost constant at the same process. This is because the torsional stiffness of the vertical beam is inversely proportional to its length.

Similarly, for a given vertical beam length, the second eigenfrequency decreases with the length of horizontal beam increasing and the first eigenfrequency keeps almost constant at the same process. This is because the bending stiffness of the vertical beam is inversely proportional to its length. Only conclusion is drawn here and the relationship curve is omitted. It is clear that the first and second eigenfrequency are mainly decided by the torsional stiffness of the vertical beam and the bending stiffness of the horizontal beam respectively.

### 3.2. Acoustic-Structural Coupled Analysis

Acoustic-structural coupled analysis which takes the fluid-structure interaction into account was also performed to characterize the dynamic behavior of the designed structure in the acoustic field so that its directivity can be proved.

The FEA model includes a fluid domain and a structural domain. The element type of the fluid domain is fluid30. Each nodes of the element has four degrees of freedom: translations in the nodal x, y and z directions and pressure. The element type of the structural domain is solid92, having three degrees of freedom at each node: translations in the nodal x, y and z directions. The analysis type in ANSYS is harmonic response analysis and the response of each square diaphragm under the excitation of incoming plane acoustic wave with different incident angle and frequency has been obtained.

For sound source location, low frequency sound signals should be used, because of great attenuation and short travel distance of high frequency signal. Then, the working frequency (also simulation frequency) will be lower than the first eigenfrequency. To simplify the expression, when we refer to phase difference (or amplitude difference), it means the phase difference (or amplitude difference) between the responses of the geometric center points of the left and the right square diaphragms.

**Figure 7.**
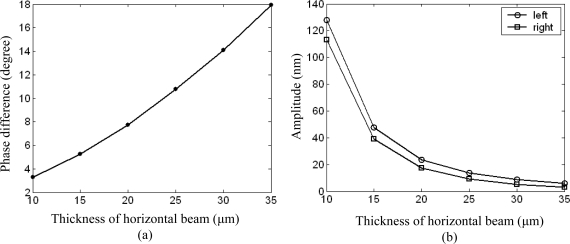
Phase difference (a) and amplitude of center point (b) versus the thickness of vertical beam.

The simulation results of the phase difference and amplitude of each center point versus the thickness of the horizontal beam are plotted for given frequency *f* = 1 kHz and incident angle *θ* = *π*/3 (the sound source is on the left side of the sensitive structure), as shown in Figure 7.

As we can see from Figure 7(a), the phase difference increases with increasing thickness of the horizontal beam, in other words, with increasing stiffness of the horizontal beam. In fact, the stiffness of the beam represents the degree of mechanical coupling between the two square diaphragms. If the beam is soft enough, the two square diaphragms respond to the sound pressure independently, the phase difference equals almost zero. If the beam is rigid enough, two square diaphragms and the connecting beam between them act like a rigid body and respond only to pressure gradient, the phase difference equals almost 180°. It can be inferred that the phase difference increases with increasing stiffness of the horizontal beam until reach its upper limit 180°. Meanwhile, the amplitude of each square diaphragm decreases with increasing beam thickness [Figure 7(b)]. This can be explained as follows: The structure’s response to acoustic pressure results in bending of the structure. As the bending stiffness increase, the displacement of each square diaphragm under pressure will decrease. Structure’s response to acoustic field, that is, the combination of the structure’s response to pressure which is dominant and response to pressure gradient, will also decrease. So a moderate stiffness beam should be chosen to acquire both larger phase difference and larger vibration amplitude of each square diaphragm for detection purpose.

**Figure 8.**
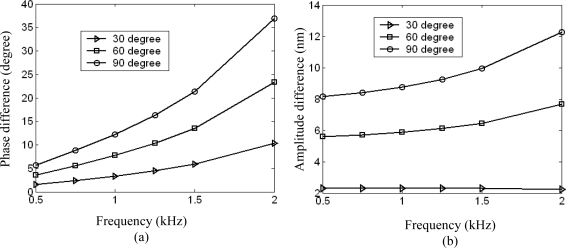
Phase difference (a) and amplitude difference (b) versus wave frequency and incident angle.

The simulation results of the phase difference or amplitude difference versus the incident angle and wave frequency are plotted, as shown in Figure 8.

As we can see from the figure, the phase difference or amplitude difference increases with increasing incident angle or frequency. This can be explained as follows: The phase difference or amplitude difference lies in structural response to the moment resulting from the pressure difference between the two square diaphragms. As we can know from acoustic principles, for harmonic acoustic wave, the pressure difference between the two acoustic signals impinging on the left and right square diaphragms increases with increasing incident angle or decreasing acoustic wavelength (which is inversely proportional to frequency). As the pressure difference increases, the moment also increases. This will result in aforementioned result. It is obvious that the phase difference or amplitude difference is effective enlarged and can be used to distinguish the direction of sound arrival.

## 4. Fabrication Process

**Figure 9.**
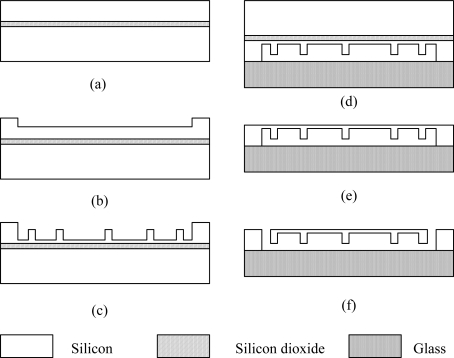
Schematic diagram of the fabrication process.

The fabrication process of the sensitive structure using silicon-on-insulator (SOI) wafer with micromachining technique is depicted in Figure 9. The device layer of the SOI wafer [Figure 9 (a)] is etched using ICP (Inductively Coupled Plasma) etching to form the steps for bonding [Figure 9 (b)]. ICP etching is a highly anisotropic etch process used to create deep, steep-sided structure. This step is followed by another etching process to fabricate the beams and stiffeners under the diaphragm [Figure 9(c)]. After that, the silicon wafer is bonded with a glass wafer through anodic bonding [Figure 9(d)]. The handle wafer and buried silicon dioxide layer of the SOI wafer is stripped by wet etching and only the device layer is remained [Figure 9(e)]. At last, slits separating the diaphragm from the surroundings are etched through ICP [Figure 9(f)], so that the sensitive structure is released and can rotate around the vertical beam.

**Figure 10.**
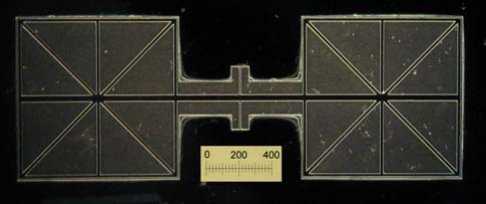
Micrograph of the acoustic pressure gradient sensitive structure.

The fabrication result of the structure is shown in Figure 10 (the length of the scale is 400 μm). This photograph is taken from the backside of the sensitive structure through the Pyrex glass bonded onto the device layer of the SOI wafer. Future work has to be carried out to further decrease the fabrication defects of the device.

## 5. Conclusions

A MEMS biomimetic acoustic pressure gradient sensitive structure for sound source localization is designed and fabricated by mimicking the mechanically coupled tympana of the parasitoid fly and conclusions can be drawn as follows:

Firstly, the structure’s response to acoustic pressure gradient is crucial to enlarge the phase difference or amplitude difference between the responses of left and right square diaphragms in the sensitive structure.
Secondly, the amplitude difference increases with increasing stiffness of the horizontal beam. A moderate stiffness beam should be chosen to acquire both larger phase difference and larger vibration amplitude of each square diaphragm for detection purpose.
Thirdly, the phase difference and amplitude difference increase with increasing incident angle or frequency, that is, the structure has directivity even when its characteristic length is much smaller than the wavelength so as to be used for sound source localization. Different incident angle will be distinguished by detecting the mechanically enlarged phase difference or amplitude difference.
